# Treatment with PPAR*α* Agonist Clofibrate Inhibits the Transcription and Activation of SREBPs and Reduces Triglyceride and Cholesterol Levels in Liver of Broiler Chickens

**DOI:** 10.1155/2015/347245

**Published:** 2015-11-25

**Authors:** Lijun Zhang, Chunyan Li, Fang Wang, Shenghua Zhou, Mingjun Shangguan, Lina Xue, Bianying Zhang, Fuxiang Ding, Dequan Hui, Aihua Liang, Dongchang He

**Affiliations:** ^1^Institute of Animal Husbandry and Veterinary Sciences, Shanxi Provincial Academy of Agricultural Sciences, Taiyuan 030031, China; ^2^Institute of Biotechnology of Shanxi University, Key Laboratory of Chemical Biology and Molecular Engineering of Ministry of Education, Taiyuan 030006, China

## Abstract

PPAR*α* agonist clofibrate reduces cholesterol and fatty acid concentrations in rodent liver by an inhibition of SREBP-dependent gene expression. In present study we investigated the regulation mechanisms of the triglyceride- and cholesterol-lowering effect of the PPAR*α* agonist clofibrate in broiler chickens. We observed that PPAR*α* agonist clofibrate decreases the mRNA and protein levels of LXR*α* and the mRNA and both precursor and nuclear protein levels of SREBP1 and SREBP2 as well as the mRNA levels of the SREBP1 (*FASN* and* GPAM*) and SREBP2 (*HMGCR* and* LDLR*) target genes in the liver of treated broiler chickens compared to control group, whereas the mRNA level of* INSIG2*, which inhibits SREBP activation, was increased in the liver of treated broiler chickens compared to control group. Taken together, the effects of PPAR*α* agonist clofibrate on lipid metabolism in liver of broiler chickens involve inhibiting transcription and activation of SREBPs and SREBP-dependent lipogenic and cholesterologenic gene expression, thereby resulting in a reduction of the triglyceride and cholesterol levels in liver of broiler chickens.

## 1. Introduction

The lipid metabolism in mammalian is regulated mainly by transcription factors including peroxisome proliferator-activated receptor alpha (PPAR*α*), liver X receptor alpha (LXR*α*), and sterol regulatory element-binding proteins (SREBPs) [1–6]. PPAR*α* is a ligand-activated transcription factor known to regulate expression of numerous genes involved in fatty acid uptake and oxidation, ketogenesis, gluconeogenesis, cholesterol catabolism, and lipoprotein metabolism [7, 8]. Transcriptional regulation of genes by PPAR*α* is mediated by forming a heterodimer with the retinoid X receptor (RXR) and subsequent binding of the PPAR*α*/RXR heterodimer to peroxisome proliferator response element (PPRE) presenting in the promoter of target genes. Ligands of PPAR*α* are fatty acids and fatty acid derivatives (eicosanoids) as well as a heterogenous group of synthetic compounds including the fibrate class of lipid lowering drugs (clofibrate, fenofibrate, bezafibrate, and gemfibrozil) [7, 8]. The lipid-lowering mechanism of fibrates involves activation of PPAR*α* in the liver leading to an upregulation of genes involved in cellular fatty acid uptake, carnitine-dependent mitochondrial fatty acid uptake, and mitochondrial and peroxisomal *β*-oxidation and, thereby, to an increased fatty acid catabolism and decreased triacylglycerol concentrations in liver and blood [7, 8]. In addition, it has been shown that activation of PPAR*α* by fibrates and oxidized fatty acids decreases the expression of genes involved in lipid synthesis and lipid uptake in the liver [9–12] indicating that the lipid-lowering effect of fibrates also involves reduction of lipid synthesis.

The LXR*α* is implicated in regulation of intracellular cholesterol levels and lipogenesis in mammals [13–15]. LXR*α* functions by forming obligate heterodimers with the retinoid X receptor (RXR) and subsequently binds to LXR response element (LXRE) within the promoters of the target genes, thereby regulating gene expression [14]. It was reported that fatty acid metabolism in rat is regulated by cross-talk between PPAR*α* and LXR*α*, in which PPARs suppress SREBP1c activation through inhibition of LXR signaling [16–18].

The SREBPs are transcription factors regulating the transcription of genes related to lipid synthesis and uptake [5, 6, 19], from which the SREBP1c isoform preferentially activates genes required for fatty acid and triacylglycerol synthesis like fatty acid synthase (*FASN*) and glycerophosphate acyltransferase, and mitochondrial (*GPAM*) [1, 20], and the SREBP2 isoform stimulates mainly genes involved in cholesterol synthesis and uptake such as 3-hydroxy-3-methylglutaryl CoA reductase (*HMGCR*) and low-density lipoprotein receptor (*LDLR*) [2, 19]. SREBPs are synthesized as inactive precursor proteins and form a complex with SREBP cleavage activating protein (SCAP), which is initially bound to the rough endoplasmic reticulum membrane by the insulin-induced genes (*INSIGs*). Activation of SREBPs involves the release of the SCAP-SREBP complex from INSIGs and its translocation to the Golgi, where the N-terminus of SREBP is cleaved by proteolysis and translocated to the nucleus where it can bind to specific sterol response elements (SREs) in the promoters of target genes, thereby activating their transcription [5, 6].

It has been shown that feeding PPAR*α* activators to rats or treatment of rat liver cells with PPAR*α* activators causes an inhibition of SREBP1 and SREBP2 activation and an upregulation of* INSIG1* and* INSIG2* [9–11] suggesting that the decreased expression of genes involved in lipid synthesis and lipid uptake in response to fibrates and oxidized fatty acids is mediated by PPAR*α*. The major sites of fatty acid synthesis are adipose tissue and the liver in animals. However, the relative contribution for the whole body lipogenesis is highly variable among species. In the pigs and ruminants adipose tissue is the main lipogenic organ with minor contribution from the liver, while in the rodents and rabbits both liver and adipose tissue are important for lipogenesis [20, 21]. However in avian species the liver is the predominant lipogenic site [20, 22–24] because its lipogenesis capacity markedly exceeds that of adipose tissue [25], indicating that an inhibitory effect of fibrates on hepatic lipid synthesis has a greater impact on lipid concentrations in birds than in other species. To our knowledge, however, it has not been shown whether fibrates inhibit the activation of SREBPs in the liver of birds. Therefore, the aim of the present study was to investigate the effect of the PPAR*α* agonist clofibrate on activation of hepatic SREBPs in birds by determining both expression levels of mRNA and protein of LXR*α* and SREBP1/SREBP2 and mRNA levels of the SREBP1 target genes* FASN* and* GPAM* and SREBP2 target genes* HMGCR* and* LDLR* as well as the mRNA levels of* INSIG1* and* INSIG2*. For this end, we performed a feeding experiment with broiler chickens that were fed either a control diet or a diet supplemented with clofibrate for 4 weeks.

## 2. Material and Methods

### 2.1. Animals Treatment

All experimental procedures were approved by the Shanxi Administration Office of Laboratory Animals (2012011035-2). A total of 48 one-day-old Arber Acres broiler chickens were used in the experiment. Until the end of the second week, all broilers were fed with a commercial starter diet (Nutrition R&D Center of Institute of Animal Husbandry and Veterinary Sciences, Shanxi Provincial Academy of Agricultural Sciences) containing 13.3 MJ metabolizable energy and 19.1% crude protein per kg diet. At the beginning of the third week, the broiler chickens were randomly assigned into two groups of 24 animals each. The animals of both groups were kept in groups of 8 birds/group in wire cages in a room with controlled temperature of 22–24°C on a 18 h light and 6 h dark cycle. The animals of the control group were fed a commercial grower diet (Nutrition R&D Center of Institute of Animal Husbandry and Veterinary Sciences, Shanxi Provincial Academy of Agricultural Sciences), whereas animals of the clofibrate group were fed the same diet supplemented with 1 g clofibrate [ethyl 2-(4-chlorophenoxy)-2-methylpropanoate] (TCI-Tokyo chemical Industry, Tokyo, Japan) per kg diet. This dose was chosen based on a recent study [26], in which a clofibrate dose of 1.5 g/kg diet was used to treat 20-week-old laying hens for 4 weeks and caused an activation of PPAR*α* in animal liver without toxic effects. Considering health and growth of broiler chickens we chose a clofibrate dose of 1 g/kg diet. For 1 kg diet 1 g clofibrate was dissolved in 25 mL sunflower oil and mixed very well with the vitamin-mineral premix, subsequently with ration of rest. The control animals received an equal volume of the vehicle. The composition of the experimental grower diets is shown in Table 1. Feed and water were supplied* ad libitum* during the entire experiment. Body weight and feed intake were recorded every week. Feed conversion ratio (FCR) was calculated by measurement of feed intake to body weight gain.

### 2.2. Sample Collection

After treatment for 4 weeks, all birds were individually weighed and then killed. Liver was excised and weighed. Aliquots of liver tissue for RNA and protein isolation as well as lipid extraction were snap-frozen in liquid nitrogen and stored at −80°C.

### 2.3. Determination of Lipid Concentrations in the Liver

For determination of triglyceride and total cholesterol concentrations in the liver, liver lipids were extracted from pooled liver tissue, with liver tissue from 3 animals contributing to each pool (about 15–20 mg from each animal for determination of triglyceride, and 10–15 mg for determination of total cholesterol), using a mixture of n-hexane and isopropanol (3 : 2, v/v) [27], and aliquots of the lipid extracts were dried and dissolved in a small volume of Triton X-100 [28]. Concentrations of triglycerides and cholesterol were determined using enzymatic reagent kits (Tissue triglyceride assay kit, catalogue number E1013 and tissue total cholesterol assay kit, catalogue number E1015, Applygen Technologies Co., Ltd., Beijing, China) following the manufacturer's protocol.

### 2.4. Total RNA Isolation and Quantitative Real-Time PCR Analysis (qPCR)

#### 2.4.1. RNA Isolation

For RNA isolation and Real-Time qPCR analysis we used the same protocol described by Keller et al. [29] with minor modifications. Briefly, total RNA was isolated from 20–30 mg of frozen liver tissue using Trizol Reagent (Shanghai Invitrogen Biotechnology Co., Ltd., China) according to the manufacturer's protocol. RNA concentration and purity were estimated by measuring the optical density (OD) at 260 and 280 nm, respectively, using NanoDrop Spectrophotometer ND-1000 (Thermo Fisher Biochemical Product (Beijing) Co., Ltd., China). RNA used for RT-PCR had an A260/A280 ratio 1.94 ± 0.05. RNA integrity and quality were evaluated by 1.2% agarose gel electrophoresis and all samples had intact bands corresponding to the 18S and 28S ribosomal RNA subunits (Supplementary Figure 1 in Supplementary Material available online at http://dx.doi.org/10.1155/2015/347245).

#### 2.4.2. cDNA Synthesis

The first-strand cDNA was synthesized using 1.2 *μ*g of total RNA, 1 *μ*L dT18 (100 pmol/*μ*L) primer (TaKaRa, Dalian, China), 1.25 *μ*L dNTP mix (10 mM) (Thermo Fisher Biochemical Product (Beijing) Co., Ltd., China), 5 *μ*L buffer (5x reaction buffer), 0.3 *μ*L M-MuLV Reverse Transcriptase (200 units/*μ*L) (Thermo Fisher Biochemical Product (Beijing) Co., Ltd., China), and x *μ*L DEPC treated water to make a 25 *μ*L final reaction volume and incubated at 42°C for 60 min, following a final inactivating step at 60°C for 10 min in Bio-Rad C1000 Touch thermal cycler PCR (Bio-Rad Laboratories, (Beijing) Co., Ltd., China).

#### 2.4.3. Primer Design and Test of Amplification Efficiency

Gene-specific primer pairs (Table 2) synthesized by TaKaRa (Dalian, China) were designed using Clone Manager Professional software 9.2. All primer pairs were designed to have melting temperature of about 60°C. The primer pairs, if possible, were designed to be located in different exons. To estimate amplification efficiency of primer, a cDNA pool from each sample was made and serial dilution for standard curve for each primer was prepared. The qRT-PCR reactions were carried out in a 0.1 mL tube (Qiagen, Germany, cat. number 981103) each with a total volume of 20 *μ*L and in a Rotor-Gene Q 2plex HRM System (Qiagen, Germany, cat. number 9001630). Each PCR mixture contained 2 *μ*L cDNA, 0.4 *μ*L each of 10 *μ*M forward and reverse primers, 10 *μ*L Maxima SYBR Green qPCR Master Mix (Thermo Fisher Biochemical Product (Beijing) Co., Ltd., China), and 7.2 *μ*L DNase/RNase free water. The qRT-PCR protocol was as follows: 3 min at 95°C, followed by 30–45 cycles, a two-step PCR consisting of 5 sec at 95°C for denaturation and 20 sec at 60°C for annealing and extension. Subsequently, melting curve analysis was performed from 50°C to 95°C to check for the presence of a single PCR product or contamination during the PCR reaction. In addition, the amplifications of specific PCR products were confirmed by performing a 2% agarose gel electrophoresis stained with GelRed Nucleic Acid Gel Stain (Biotium, (Beijing) Co., Ltd., China). After PCR running Ct (threshold cycle) values were collected by Rotorgene Software 5.0 (Qiagen, Germany) with an automated analysis and exported in Excel files. The correlation coefficient (*R* ^2^) and the slops were calculated using standard curve. The slop was used to determine the efficiency of each primer (Supplementary Table 1).

#### 2.4.4. Selection of Candidate Reference Genes

Reference genes were chosen from some published literatures in rat, pig, or cow [29–31]. Reference gene stability and the normalization factor were determined by performing GeNorm analysis described by Keller et al. [29] and Vandesompele et al. [32]. The candidate gene possessing *M*-value below 1.5 is considered as stably expressed gene. The optimal number of reference genes is determined by pairwise variation (*V*) analysis with a *V*-value below 0.15 [33]. After PCR running Ct values of reference genes were collected and then exported in Excel files. Ct values were transformed to relative quantification data using the equation 2^ΔCt^, where ΔCt = (minCt − Ct), where minCt is the lowest Ct value over a range of samples, and Ct is sample Ct. The sample with minimum expression was used as the calibrator with a set value of 1. Subsequently, 2^ΔCt^ results were used as input data for Microsoft excel-based software geNorm and normalization factors were calculated by the geNorm software. The *M*-value and *V*-value as well as ranking reference genes for stability of expression were reported as in the figure by geNorm output (Supplementary Figures 2 and 3). Based on the *M*-values and *V*-values, out of six tested potential reference genes including* MDH1*,* RPL13*,* GAPDH*,* YWHAZ*,* ATP5B*, and* TOP1*, the five reference genes* MDH1*,* RPL13*,* GAPDH*,* YWHAZ*, and* ATP5B* with *M* < 1.5 and *V*5/*V*6 = 0.154, which was near the proposed cutoff value of 0.15, were used to calculate a gene expression normalization factor for each sample.

#### 2.4.5. Data Analysis

After PCR running the relative quantification of target genes was performed using 2^ΔCt^ method described above and then normalized by the normalization factor using geNorm. The qRT-PCR data for each gene prior to statistical analysis were normalized to the control by dividing each data point by the mean of the control group. This resulted in a mean of 1 for the control and an expression ratio for the treated group compared to the control group.

### 2.5. Immunoblot Analysis

Nuclear extracts were prepared from 100 mg pooled liver tissue, with liver tissue from 3 animals contributing to each pool (about 30–40 mg from each animal), using Nuclear Extract Kit (Active Motif, (Shanghai) Co., Ltd., China) according to the manufacturer's protocol. Protein content was determined by the bicinchoninic acid protein assay kit (TaKaRa, Dalian, China) and BSA as standard. Proteins were separated by SDS-PAGE and electrotransferred to nitrocellulose membranes (Bio-Rad Laboratories, (Beijing) Co., Ltd., China). The blots were incubated overnight at 4°C with primary antibodies against rabbit polyclonal SREBP1 (1 : 500, Santa Cruz, USA), SREBP2 (1 : 500, Santa Cruz, USA), and LXR*α* (1 : 500, Affinity Bioreagents, USA), as well as mouse monoclonal *β*-actin (1 : 10.000, Abcam, Cambridge, UK) as internal control for normalization. The membranes were washed and then incubated with a horseradish peroxidase-conjugated secondary anti-rabbit-IgG or anti-mouse IgG antibody (1 : 10.000 Santa Cruz, USA) at room temperature. Afterward, blots were developed using ECL Plus (Bio-Rad Laboratories, (Beijing) Co., Ltd., China). Band intensities were evaluated densitometrically using ChemiDoc MP Image Lab System and Image Lab Software 5.1 according to the manufacturer's guideline (http://www.bio-rad.com/webroot/web/pdf/lsr/literature/10022469.pdf) (Bio-Rad Laboratories, (Beijing) Co., Ltd., China).

### 2.6. Statistics Analysis

Data were expressed as means  ±  SEM (standard error of mean). Statistical analysis was performed using SAS 9.1.3 statistical software (SAS Institute, Inc., 2001). Data of experiment were analyzed for normality of distribution (Anderson-Darling test). Because all data showed a normal distribution, one-way ANOVA was applied to evaluate the effect of treatment. Means of the treatment (clofibrate group) were compared with the control group by Student's *t*-test. Significant difference was declared with a *P* < 0.05.

## 3. Results

### 3.1. Body Weights, Feed Intake, and Feed Conversion Ratio

Feeding of the diet with clofibrate did not reduce performance characteristics of broiler chickens. Initial body weight of the experimental animals (IBWE) and final body weights (FBW), average daily feed intake (ADF), and feed conversion ratio (FCR) did not differ between the control group and the clofibrate group (IBWE: 314 ± 10 versus 319 ± 11 g; FBW: 1946 ± 45 versus 1966 ± 43 g; ADF: 123 ± 2 versus 108 ± 2 g; FCR: 1.93 ± 0.06 versus 2.06 ± 0.04; *n* = 24/group).

### 3.2. Concentrations of Triglyceride and Cholesterol in the Liver

The concentrations of triglyceride and cholesterol were significantly lower in the liver of broiler chickens treated with clofibrate than that of the control group (Table 3).

### 3.3. Relative mRNA Concentrations of PPAR*α* and the PPAR*α* Target Gene CPT1A in the Liver

**Figure 1:**
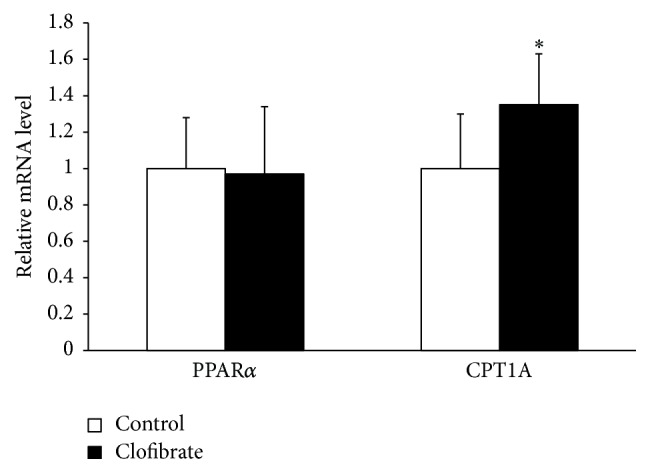
mRNA level relative to control of* PPARα* and its target gene* CPT1A* in the liver of broiler chickens fed diets without (control) or with 0.1% clofibrate for 4 weeks. Values are means ± SEM, *n* = 24/group.

To evaluate activation of hepatic PPAR*α* by clofibrate, we determined the mRNA level of the classical PPAR*α* target gene* CPT1A* in the liver. As shown in Figure 1, the mRNA level of* CPT1A* in the liver was about 35% greater in the clofibrate group than in the control group (*P* < 0.05) indicating activation of hepatic PPAR*α* by clofibrate. The mRNA level of* PPARα* in the liver did not differ between broiler chickens of the control group and the clofibrate group.

### 3.4. Relative mRNA Concentrations of* SREBFs* and Their Target Genes in the Liver

**Figure 2:**
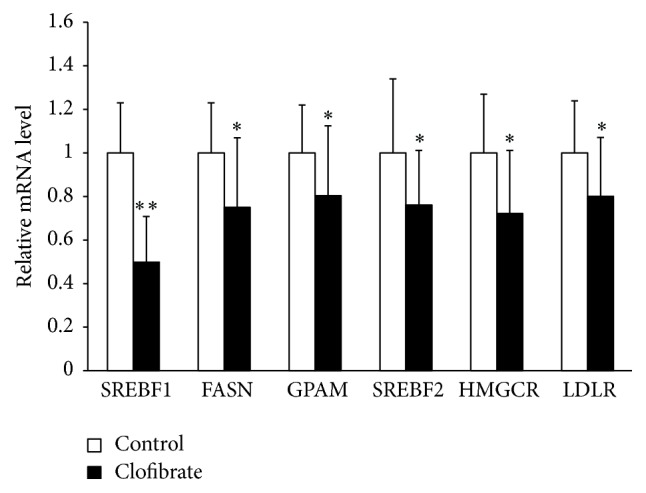
mRNA level relative to control of* SREBF1*,* SREBF2*, and SREBP1 (*FASN*,* GPAM*) and SREBP2 (*HMGCR*,* LDLR*) target genes in the liver of broiler chickens fed diets without (control) or with 0.1% clofibrate for 4 weeks. Values are means ± SEM, *n* = 24/group.

To investigate whether activation of hepatic PPAR*α* is accompanied by a reduced expression of genes involved in lipid synthesis and uptake in the liver, we determined mRNA levels of* SREBFs* and SREBP target genes. As illustrated in Figure 2, mRNA levels of* SREBF1* and SREBP1 target genes (*FASN*,* GPAM*) and* SREBF2* and SREBP2 target genes (*HMGCR*,* LDLR*) in the liver of broiler chickens were approximately 20% to 50% less in the clofibrate group than in the control group (*P* < 0.05).

### 3.5. Relative Protein Levels of Precursor and Nuclear SREBPs in the Liver

**Figure 3:**
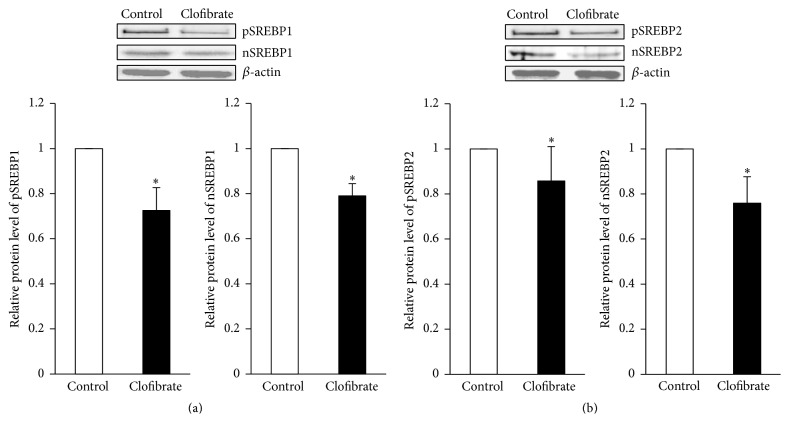
Protein levels relative to control of precursors and nuclear forms of SREBP1 and SREBP2 in the liver of broiler chickens fed diets without (control) or with 0.1% clofibrate for 4 weeks. Representative immunoblots specific to precursors and nuclear SREBP1 (a) and SREBP2 (b) and *β*-actin as internal control are shown for one pool per group; immunoblots for the other pools revealed similar results. Values are means ± SEM, *n* = 3 pools/group with each pool representing 3 animals.

In order to explain the reduced expression of SREBP target genes in the liver of clofibrate-treated broiler chickens, we determined the protein levels of precursor and the transcriptionally active nuclear SREBPs in the liver. In line with the decreased mRNA levels of SREBP target genes in the liver, we found that the protein levels of precursor and nuclear SREBP1 (Figure 3(a)) and SREBP2 (Figure 3(b)) in the liver of broiler chickens were decreased (about pSREBP1 28%, nSREBP1 21%; pSREBP2 14%, nSREBP2 24%) in the clofibrate group compared to the control group (*P* < 0.05).

### 3.6. Relative mRNA Levels of* INSIG1* and* INSIG2* in the Liver

**Figure 4:**
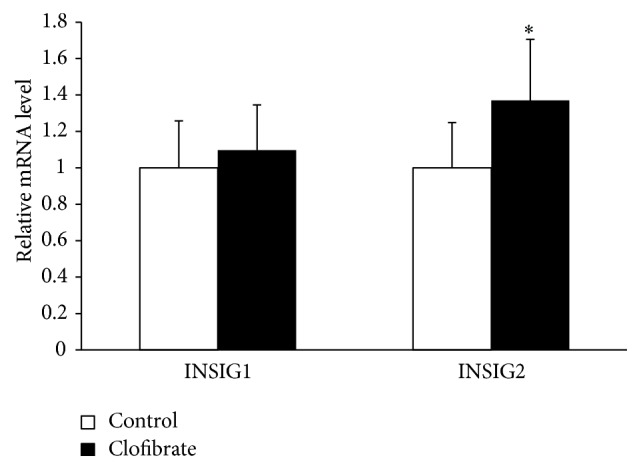
mRNA level relative to control of* INSIG1* and* INSIG2* in the liver of broiler chickens fed diets without (control) or with 0.1% clofibrate for 4 weeks. Values are means ± SEM, *n* = 24/group.

To study whether the reduced activation of SREBPs by clofibrate in the liver of broiler chickens involves upregulation of* INSIGs*, we determined mRNA levels of* INSIG1* and* INSIG2* in the liver. As demonstrated in Figure 4, the relative mRNA level of* INSIG2* in the liver was about 40% greater in the clofibrate group than in the control group (*P* < 0.05), whereas that of* INSIG1* did not differ between the two groups.

### 3.7. Relative mRNA and Protein Level of LXR*α* in the Liver

**Figure 5:**
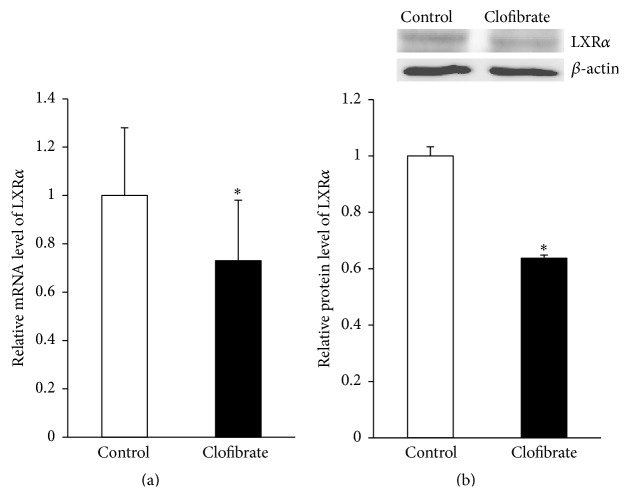
mRNA and protein level relative to control of LXR*α* in the liver of broiler chickens fed diets without (control) or with 0.1% clofibrate for 4 weeks. (a) Bars represent means ± SEM (*n* = 24/group) and are expressed mRNA level relative to the control group. (b) Representative immunoblot specific to nuclear LXR*α* and *β*-actin as internal control is shown for one pool per group; immunoblots for the other pools revealed similar results. Values are means ± SEM, *n* = 3 pools/group with each pool representing 3 animals.

In order to explain the reduced expression of SREBP target genes in the liver of clofibrate-treated broiler chickens, we finally determined mRNA and nuclear protein levels of LXR*α* in the liver. We observed that both mRNA (Figure 5(a)) and nuclear protein levels of LXR*α* (Figure 5(b)) in the liver of broiler chickens were decreased (about 25% and 35%, resp.) in the clofibrate group compared to the control group (*P* < 0.05).

## 4. Discussion

In the present study we investigated the regulatory mechanism of the triglyceride- and cholesterol-lowering effect of the PPAR*α* agonist clofibrate in broiler chickens, a species in which the liver is the predominant site of lipogenesis. We found that clofibrate did not influence the final body weight and feed conversion ratio between the control and treatment groups but lowered the concentrations of triglyceride and cholesterol in the liver of broiler chickens. We also observed that mRNA and protein levels of LXR*α* and mRNA and both precursor and nuclear protein levels of SREBP1c and SREBP2, which are the master regulators of genes involved in lipid synthesis and uptake, as well as* the* expression of SREBP1c and SREBP2 target genes (*FASN*,* GPAM*,* HMGCR*, and* LDLR*) were clearly decreased in treatment group. The similar effects were found in the liver of rats and rat liver cells by treatment with PPAR*α* activators [9–11]. Numerous studies reported that SREBPs were regulated at multiple levels, namely, at the mRNA, precursor, or mature protein levels [34–37]. SREBP2 controls cholesterolgenic genes primarily by affecting proteolytic processing with only minor changes in the level of mRNA, whereas SREBP1c regulates lipogenic enzymes mainly by self-regulating its own transcription level due to the presence of SRE in promoter of* SREBF1*, or rather by changing mRNA level of* SREBF1* inhibiting the proteolytic activity to cleave SREBP1c precursor into its nuclear form, indicating that SREBP1 is regulated in a different fashion than SREBP2 [35–37]. The present study demonstrated that the abundance of the precursor SREBP1c and the abundance of its nuclear form were decreased in a proportional way by the clofibrate treatment. This indicates that the decrease in lipogenesis observed is mostly due to a decrease in the overall transcription of* SREBF1*. Peterson et al. [34] demonstrated that a short term (48 h) treatment with* trans*-10,* cis*-12 CLA reduces lipid synthesis in bovine mammary epithelial cells through inhibition of proteolytic activation of SREBP1 and subsequent reduction in transcriptional activation of lipogenic genes. A longer-term treatment (4 weeks) with clofibrate in present study would possibly lead to a reduced abundance of SREBP1c mRNA and precursor protein. However, the mechanism by how the clofibrate lower* SREBF1* mRNA levels in liver of broiler chickens remains to be explored. Taken together, these findings suggest that SREBP1c represses lipogenic genes such as* FASN* and* GPTM* and inhibits lipogenesis and lipid uptake in the liver of broiler chickens by a mechanism involving reduction of SREBP1 transcription and activation contributing to lower lipids levels in liver of broiler chickens.

Despite reporting in earlier studies that PPAR*α* activation is accompanied by an inhibition of SREBP-dependent gene expression, it is currently unknown how PPAR*α* activation mediates this effect. It has been identified that mouse and human* SREBF1c* gene is a direct LXR*α* target gene with two LXR response elements (LXREs) found in the* SREBF1c* promoter region [38–40]. This indicates that the expression of* SREBF1c* is regulated by LXR*α*. LXR*α*, like PPAR*α*, functions usually as obligate heterodimer with retinoid X receptor (RXR*α*) which regulates the transcription of their target genes by binding to LXRE of target promoter. A recent study has demonstrated that overexpression of* PPARα* and treatment with PPAR*α* agonist both enhance binding of PPAR*α* to RXR*α* and decrease the amount of LXR/RXR heterodimers, leading to suppression of LXR*α* ligand-activated* SREBF1c* expression in rat primary hepatocyte cultures and mouse liver [18]. This suggests that the mechanism for PPAR*α* inhibition of LXR*α*-mediated transcriptional activity of* SREBF1c* could be at RXR competition between PPAR*α* and LXR*α*. Besides, PPAR*α* can heterodimerize with LXR*α* and results in interference of LXR/RXR formation and inhibition of* SREBF1c* promoter activation [41]. Thus, our results suggest that decreased expression of nuclear LXR*α* by clofibrate may contribute to the lipid-lowering effect of PPAR*α* by inhibiting LXR-dependent* SREBF1c* transcription in the liver of broiler chickens. However, more detailed promoter studies, for example, using reporter gene or gel-shift assays, will be needed to determine if the broiler* SREBF1c* gene is also a target gene of LXR*α*.

Studies reported that SREBP2 controls cholesterol synthesis through cleavage of the membrane-bound precursor protein to liberate its nuclear active form in the nucleus [36, 37]. Convincing evidence has been provided that PPAR*α* activator WY 14,643, a potent PPAR*α* ligand, decreases hepatic cholesterol concentration in wild type mice, but not in PPAR*α* null mice, by alteration in membrane fatty acid composition that influenced SREBP activation, suggesting that PPAR*α* plays an important role to control SREBP2 activity and hepatic cholesterol biosynthesis [42]. The present study demonstrate that the nuclear protein level of SREBP2 is markedly less than precursor protein level by the clofibrate treatment indicating that SREBP2 controls cholesterol synthesis at the cleavage system. In contrast to our results the studies reported that the expression levels of mRNAs and proteins which involved in cholesterol biosynthesis were increased after WY 14,643 treatment in the liver of wild type mice, but in fact these increases seem not to be associated with hepatic de novo cholesterologenesis; thus, the present study supported the observations in mice that PPAR*α* agonist treatment does not lead to a stimulation of the hepatic cholesterol synthesis but rather decreases it [42, 43]. We have also found in the present study that clofibrate slightly increased hepatic expression of* INSIG1* and significantly that of* INSIG2*. In line with this, recent studies revealed that transcription of* INSIG1* in rat liver and* INSIG1* and* INSIG2* in rat Fao cells was increased by treatment with PPAR*α* agonist WY-14,643 [10]. Similarly, rats administered an oxidized dietary fat, like frying oil, which is known to cause strong activation of hepatic PPAR*α* [44], were found to have increased mRNA levels of* INSIG1* and* INSIG2* in the liver [9]. Both, INSIG1 and INSIG2 are responsible for retaining the precursor forms of SREBP1 and SREBP2 within the endoplasmic reticulum thereby inhibiting the proteolytic processing of SREBPs in the Golgi [45, 46]. Thus, it can be proposed that the upregulation of* INSIGs* by clofibrate causes an inhibition of the release of the SCAP-SREBP complex from INSIGs and its translocation to the Golgi, where proteolytic processing (activation) of SREBPs occurs. PPAR*α* and other PPAR isoforms (PPAR*γ*, PPAR*δ*/*β*) are known to stimulate transcription of target genes through binding as a complex with retinoic acid-X receptor to specific DNA sequences, called PPREs, in the regulatory region of target genes [8, 47, 48]. Interestingly, it has been recently shown that the human* INSIG1* gene contains a functional PPRE, which is regulated by both, PPAR*δ* [49] and PPAR*γ* [50]. Moreover, it was shown that adenovirus induced-overexpression of* PPARδ* causes induction of* INSIG1* and suppression of SREBP1 activation and lipogenesis in the liver of obese diabetic mice [49] indicating that upregulation of* INSIGs* by clofibrate may also explain inhibition of SREBP-dependent gene expression and lipogenesis in the liver of broiler chickens. Although it remains to be shown whether the chicken genes encoding* INSIG1* or* INSIG2* are also regulated by PPAR*α*, it is well-known that the functional PPREs of many PPAR target genes are regulated by all three PPAR isotypes. The* CPT1A* gene, for instance, which was used as an indicator to assess activation of PPAR*α* by clofibrate in the present study, is known to possess a functional PPRE in its promoter which is bound by PPAR*α*, PPAR*γ*, and PPAR*δ*/*β* target genes in broilers. However, future studies using reporter gene and gel-shift assays have to clarify whether or not the genes encoding* INSIGs* are PPAR target genes.

In conclusion, the results of the present study demonstrated that PPAR*α* agonist clofibrate lowers the triglyceride concentration in broiler liver by reducing transcription and activation of SREBP1 and by repressing LXR*α*-mediated transcriptional activity of SREBP1, which subsequently reduced lipogenic gene expression of* FASN* and* GPTM*, whereas PPAR*α* agonist clofibrate decreases cholesterol concentration in broiler liver by upregulating the expression of* INSIG2* that inhibits proteolytic cleavage and activation of SREBP2, subsequently reducing SREBP2-dependent gene expression of* LDLR* and* HMGAR*, thereby resulting in the decrease of synthesis capacity of triglyceride and cholesterol in the broiler liver.

## Supplementary Material

To support the findings of this manuscript the additional information about RT-qPCR method were added in supplementary materials including:
Supplementary Figure 1: Analysis of RNA integrity and quality.
Supplementary Figure 2: Average expression stability values (M) and ranking of candidate reference genes.
Supplementary Figure 3: Determination of the optimal number of control genes for normalization.
Supplementary Table 1: Quantitative real-time PCR performance data.

## Figures and Tables

**Table 1:** Composition of the experimental grower diets.

	Control group	Treatment group
Components (g/kg)		
Maize	630	629
Fish meal	25	25
Wheat middlings	30	30
Soybean meal 44%	260	260
Sunflower oil	25	25
Limestone powder	10	10
Calcium hydrogen phosphate	14.8	14.8
Sodium chloride	2.5	2.5
Vitamin-mineral mix^1^	1.2	1.2
DL-methionine	1	1
Choline chloride 50%	0.5	0.5
Clofibrate	—	1.0
Crude nutrients and energy content		
Dry matter (%)	89.84	89.84
Metabolizable energy (MJ/kg)	13.3	13.3
Crude protein (%)	19.1	19.1
Crude fat (%)	5.5	5.5
Linoleic acid (%)	2.0	2.0
Crude fibre (%)	2.6	2.6
Calcium (%)	0.98	0.98
Available phosphorus (%)	0.49	0.49

^1^The vitamin-mineral mix provided per kg of diet: 3.3 mg retinol, 0.033 mg cholecalciferol, 13.34 mg D-*α*-tocopheryl acetate, 0.5 mg menadione, 2.2 mg thiamine, 6.6 mg riboflavin, 5.5 mg pyridoxine, 0.01 mg cobalamin, 13.5 mg pantothenate, 0.2 mg biotin, 1.0 mg folic acid, 44 mg nicotinic acid, 350 mg magnesium, 9.6 mg copper, 100 mg iron, 108 mg manganese, 88 mg zinc, 0.23 mg selenium, and 0.4 mg iodine.

**Table 2:** Characteristics and performance data of the primers used for reference gene-stability measure *M* and quantitative real-time PCR analysis.

Gene name	Primer sequence (forward, reverse)	Product size (bp)	NCBI GenBank
Reference genes			
* ATP5B*	GGTGTGCAGAAGATCCTTCA, GATCTGCTTGAAGCCCTTGA	216	NM_001031391
* TOP1*	GCATCATGCCAGAAGACATC, CTTGAGCTAGGGTTCAACATT	175	NM_205110
* MDH1*	GCATGGAGAGGAAGGATTTG, GAAGTCACACCAAGCTTCAG	241	NM_001006395
* RPL13*	GTTCGTGCTGGCAGAGGAT, GACAGCTGAGTTGCCATCTT	245	NM_204999
* YWHAZ*	TGTGGAGCAATCACAACAGG, GTTGTCTCTCAGTAACTGCAT	250	NM_001031343
* GAPDH*	CACTTCAAGGGCACTGTCAA, CTCATGGTTGACACCCATCA	252	NM_204305
Target genes			
* PPARα*	GGAGTTTAAGTGACCGCTCT, CTGATCCATCAGATCCTGGA	230	NM_001001464
* CPT1A*	ACTCTCCAGCACGTGAAAGA, CCTGCAGTAAGAGCTGCTAA	118	NM_001012898
* SREBF1*	ACCTGGCAGCCAAGGCAT, GACTCAGCCATGATGCTTCT	168	NM_204126
* FASN*	CTCCTTGAAGGTGGTTTGCA, CCTCCATGTTTCCTGCTTTC	219	NM_205155
* GPAM*	GTTGAGACAGCAGCAGTTTTT, CCTTCAATTATGCGATCGTAG	177	XM_421757
* SREBF2*	CATTCTGACCACAATGCCAG, GGTCCTTCAGCTCAATGATC	176	AJ414379
* HMGCR*	GCTTAGCCTTTCTCCTTGCT, CCAGATTGTTTCCTGCAGCA	250	XM_422225
* LDLR*	CACTCAGTGCCACCATTTGG, CTGCGACGGAACGTCCAAG	203	NM_204452
* INSIG1*	CCAACAATGTCCAGCTGTCC, CACCATTATATACAAGGAACTG	147	NM_001030966
* INSIG2*	GGATTTTGCCAACAATATCCAG,TGTAGACCAGAAGCTGTGACA,	149	NM_001031261
* LXRα*	CCAAGATGCTGGGAAATGAAGC, ATATACATGTCCATCTCACAC	193	NM_204542

Sterol regulatory element binding transcription factors 1 and 2 (*SREBF1* and *SREBF2*); peroxisome proliferator-activated receptor alpha (*PPARα*); carnitine palmitoyltransferase 1A (*CTP1A*); fatty acid synthase (*FASN*); glycerol-3-phosphate acyltransferase, mitochondrial (*GPAM*); 3-hydroxy-3-methylglutaryl CoA reductase (*HMGCR*), low-density lipoprotein receptor (*LDLR*); Liver X-Receptor alpha (*LXRα*); insulin induced genes 1 and 2 (*INSIG1* and *INSIG2*); ATP synthase, H+ transporting, mitochondrial F1 complex, beta polypeptide (*ATP5B*); topoisomerase (DNA) I (*TOP1*); malate dehydrogenase 1, NAD (soluble) (*MDH1*); ribosomal protein L13 (*RPL13*); tyrosine 3-monooxygenase/tryptophan 5-monooxygenase activation protein, zeta polypeptide (*YWHAZ*); glyceraldehyde-3-phosphate dehydrogenase (*GAPDH*).

**Table 3:** Concentrations of triglycerides and cholesterol in the liver of broiler chickens fed diets without (control) or with 0.1% clofibrate for 4 weeks.

Lipid	Control	Clofibrate
Triglycerides (*µ*mol/g)	74.6 ± 4.56	35.5 ± 2.83^*∗*^
Cholesterol (*µ*mol/g)	11.2 ± 0.40	7.5 ± 0.98^*∗*^

Values are means ± SEM, *n* = 3 pools/group with each pool representing 3 animals.

^*∗*^Significantly different from control (*P* < 0.05).
