# Supplementary material for: Treatment with PPARα Agonist Clofibrate Inhibits the Transcription and Activation of SREBPs and Reduces Triglyceride and Cholesterol Levels in Liver of Broiler Chickens
Source: PPAR Res. 2015 Nov 25;2015:347245. doi: 10.1155/2015/347245 (PMC4674622; doi:10.1155/2015/347245)
Supplement: Supplementary file 1 — To support the findings of this manuscript the additional information about RT-qPCR method were added in supplementary materials including: Supplementary Figure 1: Analysis of RNA integrity and quality. Supplementary Figure 2: Average expression stability values (M) and ranking of candidate reference genes. Supplementary Figure 3: Determination of the optimal number of control genes for normalization. Supplementary Table 1: Quantitative real-time PCR performance data. [file 347245.f1.zip › 347245.f1/Supplementary Legends and Table.pdf]

Supplementary Legends

Figure 1 Total RNA quality assessment on the basis of 18S and 28S rRNA**.** The GelRed staining pattern of intact total RNA shows clearly defined 18S and 28S ribosomal RNA bands (A control group, lanes 1-24 and B clofibrate group, lanes 1-22 und 23-24). Gels were imaged with a standard 300 nm UV transilluminator and photographic detection using ChemiDoc™ XRS+ (Bio-Rad Laboratories, (Beijing) Co., Ltd., China).

Figure 2 Average expression stability values (M) and ranking of candidate reference genes based on geNorm calculations. The genes with the least stable expression are on the left, and the most stable genes are on the right.

Figure 3 geNorm pairwise variation (V_n/V_{n+1}) analysis to determine optimal number of reference genes for normalization in RT-qPCR reaction.

Supplementary Table

Table 1. Quantitative real-time PCR performance data.

Gene name	Slope	R^{2*}	Efficiency[#]
ATP5B	-0.24	0.997	1.77
TOP1	-0.26	0.907	1.81
MDH1	-0.27	0.965	1.87
RPL13	-0.30	0.995	1.98
YWHAZ	-0.29	0.990	1.99
GAPDH	-0.23	0.998	2.01
PPARα	-0.29	0.998	1.99
CPT1A	-0.37	0.958	2.30
SREBF1	-0.27	0.998	1.88
FASN	-0.29	0.990	1.97
GPAM	-0.27	0.992	1.87
SREBF2	-0.28	0.997	1.93
HMGCR	-0.26	0.997	1.82
LDLR	-0.33	0.993	2.15
INSIG1	-0.32	0.954	2.09
INSIG2	-0.29	0.991	1.99
LXRα	-0.33	0.987	2.17

[*] R^2 Coefficient of determination of the standard curve.

[#] Efficiency The efficiency is determined by [10^{-slope}].
