# Supplementary figures and images for: Treatment with PPARα Agonist Clofibrate Inhibits the Transcription and Activation of SREBPs and Reduces Triglyceride and Cholesterol Levels in Liver of Broiler Chickens
Source: PPAR Res. 2015 Nov 25;2015:347245. doi: 10.1155/2015/347245 (PMC4674622; doi:10.1155/2015/347245)

## Slide 1
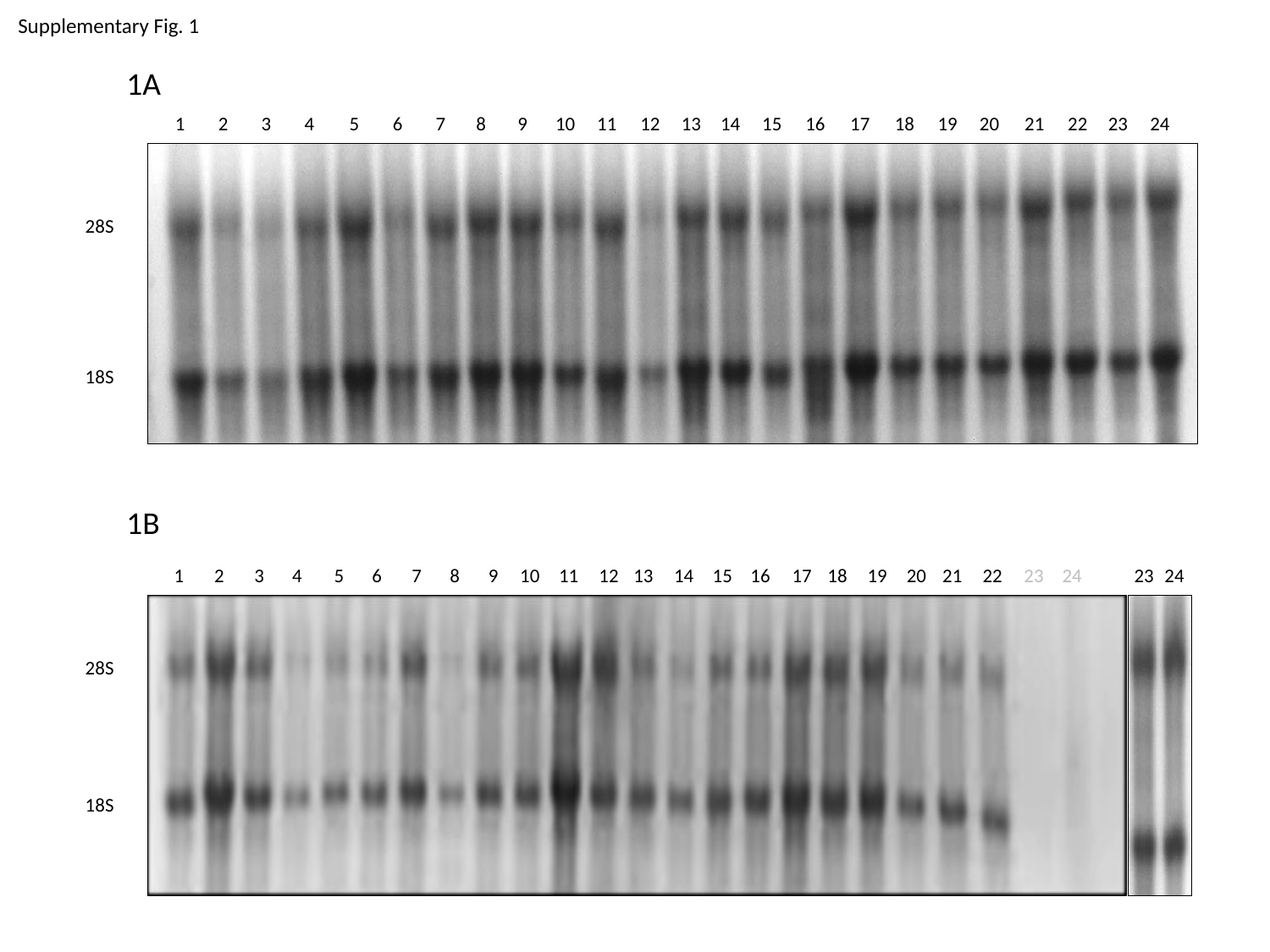

Supplementary Fig. 1
1A
1
2
3
4
5
6
7
8
9
10
11
12
13
14
15
16
17
18
19
20
21
22
23
24
28S
18S
1B
1
2
3
4
5
6
7
8
9
10
11
12
13
14
15
16
17
18
19
20
21
22
23
24
23
24
28S
18S

Supplement: Supplementary file 1 — To support the findings of this manuscript the additional information about RT-qPCR method were added in supplementary materials including: Supplementary Figure 1: Analysis of RNA integrity and quality. Supplementary Figure 2: Average expression stability values (M) and ranking of candidate reference genes. Supplementary Figure 3: Determination of the optimal number of control genes for normalization. Supplementary Table 1: Quantitative real-time PCR performance data. [file 347245.f1.zip › 347245.f1/Supplementary Fig. 1.pptx]

## Slide 1
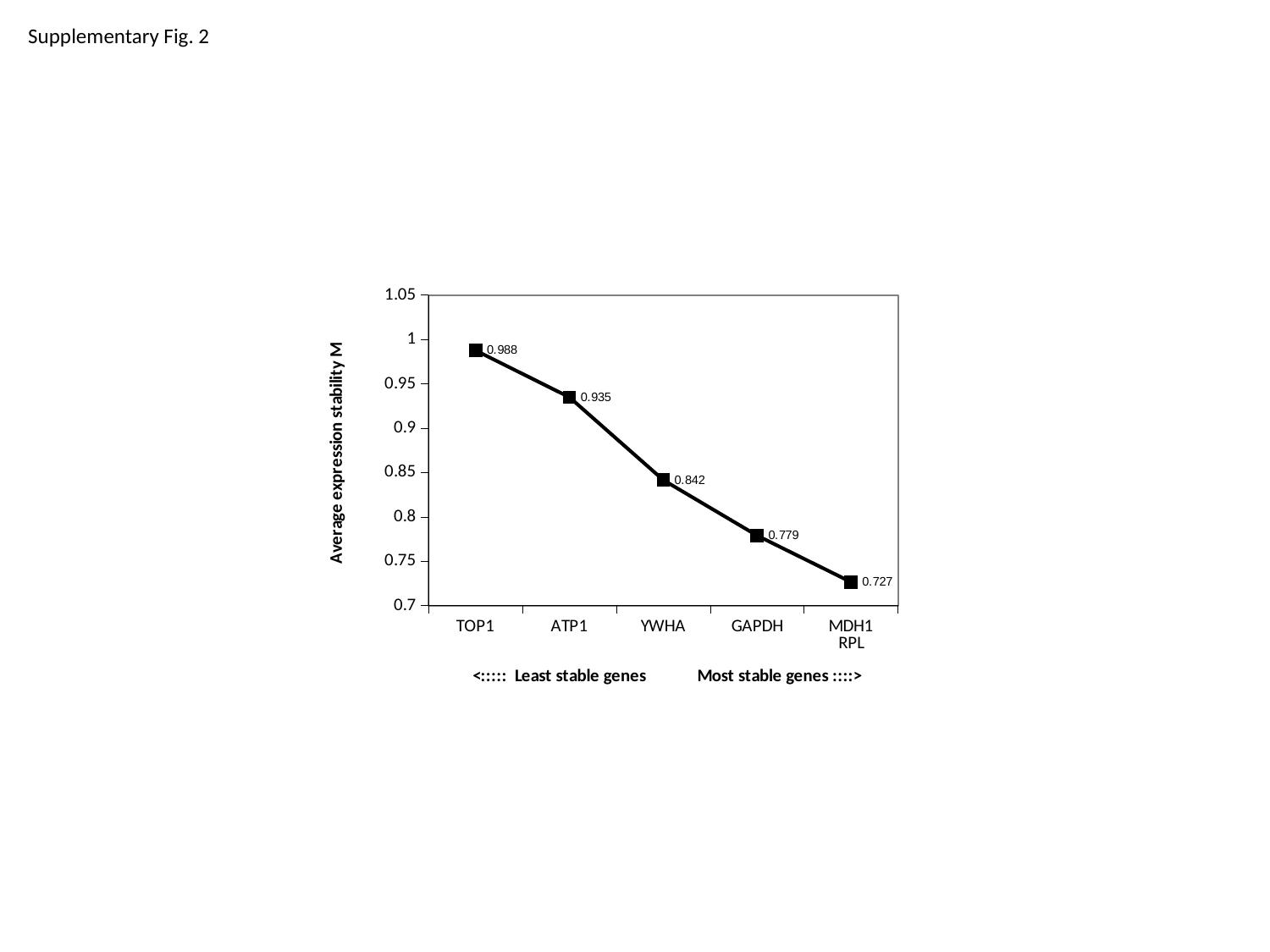

Supplementary Fig. 2
### Chart
| Category | |
|---|---|
| TOP1 | 0.9875883101296107 |
| ATP1 | 0.934900922372085 |
| YWHA | 0.8416369620909883 |
| GAPDH | 0.7790244204387324 |
| MDH1
RPL | 0.7266689935108029 |

Supplement: Supplementary file 1 — To support the findings of this manuscript the additional information about RT-qPCR method were added in supplementary materials including: Supplementary Figure 1: Analysis of RNA integrity and quality. Supplementary Figure 2: Average expression stability values (M) and ranking of candidate reference genes. Supplementary Figure 3: Determination of the optimal number of control genes for normalization. Supplementary Table 1: Quantitative real-time PCR performance data. [file 347245.f1.zip › 347245.f1/Supplementary Fig. 2.pptx]

## Slide 1
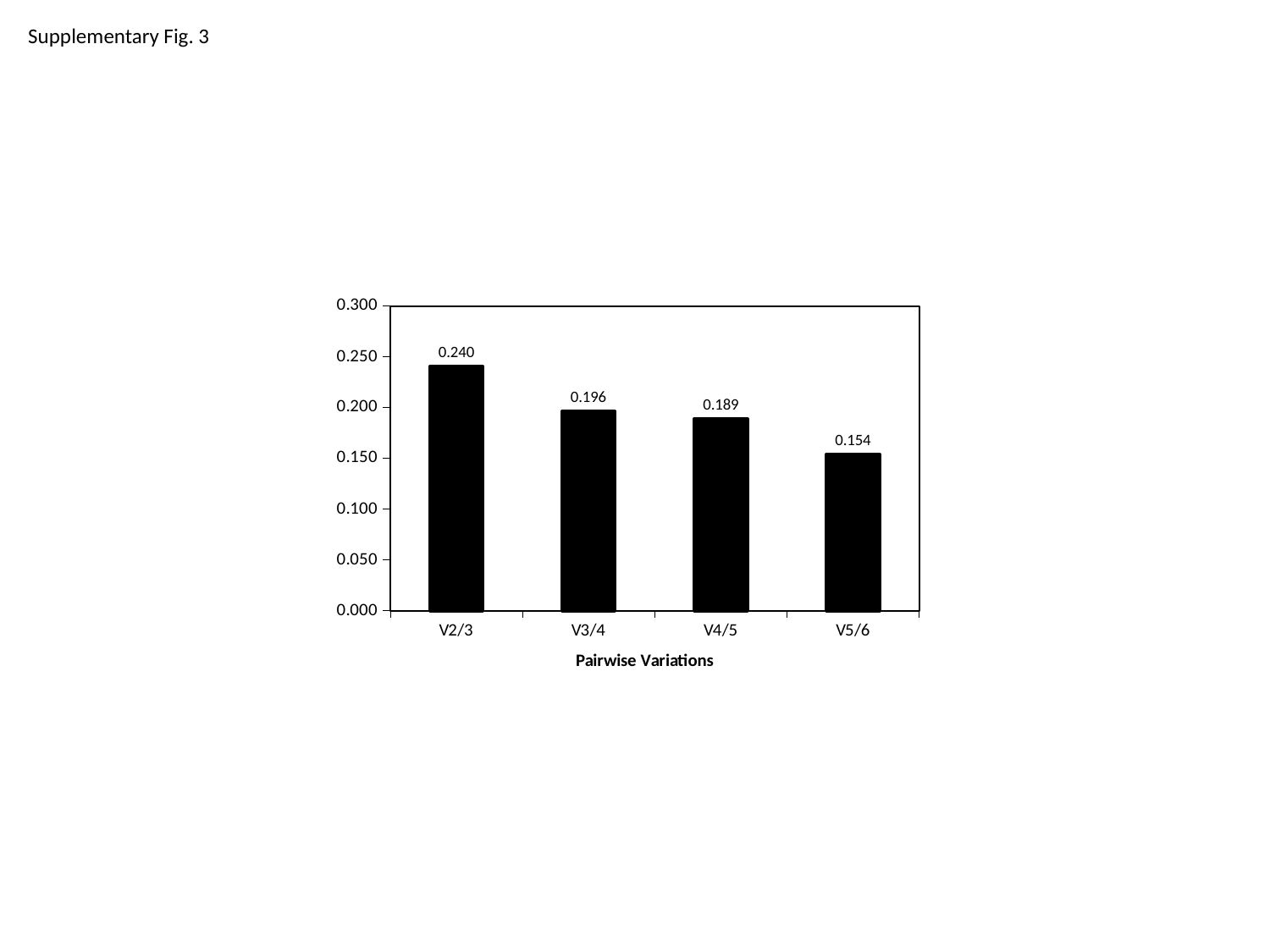

Supplementary Fig. 3
### Chart
| Category | |
|---|---|
| V2/3 | 0.24023794824356481 |
| V3/4 | 0.19603154930419966 |
| V4/5 | 0.18890416676394345 |
| V5/6 | 0.15371830920486235 |

Supplement: Supplementary file 1 — To support the findings of this manuscript the additional information about RT-qPCR method were added in supplementary materials including: Supplementary Figure 1: Analysis of RNA integrity and quality. Supplementary Figure 2: Average expression stability values (M) and ranking of candidate reference genes. Supplementary Figure 3: Determination of the optimal number of control genes for normalization. Supplementary Table 1: Quantitative real-time PCR performance data. [file 347245.f1.zip › 347245.f1/Supplementary Fig. 3.pptx]
